# Neurobiological Mechanisms of Enhanced Pain-Relieving Transcutaneous Electrical Nerve Stimulation via Visuo-Tactile Stimulation in Immersive Virtual Reality: Randomized Controlled Trial

**DOI:** 10.2196/63137

**Published:** 2025-03-19

**Authors:** Chenxi Wang, Lanqi Gao, Chuan Zhang, Jun Li, Jixin Liu

**Affiliations:** 1 School of Life Science and Technology Xidian University Xi'an China; 2 Department of Radiology Affiliated Hospital of North Sichuan Medical College Nanchong China

**Keywords:** pain, pain-relieving, transcutaneous electrical nerve stimulation, virtual reality, electroencephalography

## Abstract

**Background:**

Enhancing the effectiveness of current pain relief strategies is a persistent clinical challenge. Although transcutaneous electrical nerve stimulation (TENS) is used in various painful conditions, its effectiveness may decline over time, requiring additional pain management strategies. Immersive virtual reality (VR) with personalized visuo-tactile stimulation has demonstrated analgesic properties. Nevertheless, whether visuo-tactile stimulation can enhance the pain-relieving outcomes of TENS and its underlying neurophysiological mechanisms remains largely unknown.

**Objective:**

The study aims to investigate whether the integration of visuo-tactile stimulation with TENS can enhance the pain-relieving outcomes of TENS alone, and we also aim to explore the brain mechanisms underlying the analgesic effect of this integrated intervention.

**Methods:**

In this study, 75 healthy participants were enrolled and randomly assigned to 1 of 3 groups: congruent TENS-VR (TENS-ConVR) and 2 control groups (incongruent TENS-VR [TENS-InVR] and TENS alone). In the context of TENS-ConVR, we combined TENS and VR by connecting TENS-induced paresthesia with personalized visual bodily feedback. The visual feedback was designed to align with the spatiotemporal patterns of the paresthesia induced by TENS. A pain rating task and a 32-channel electroencephalography were applied.

**Results:**

Two-way ANOVAs showed that TENS-ConVR exhibited a statistically greater reduction in pain rating (*F*_1,48_=6.84; *P*=.01) and N2 amplitude (*F*_1,48_=5.69; *P*=.02) to high-intensity pain stimuli before and after stimulation than TENS alone. The reduction of brain activity was stronger in participants who reported stronger pain-relieving outcomes. TENS-ConVR reduced the brain oscillation in the gamma band, whereas this result was not found in TENS alone.

**Conclusions:**

This study observed that combining TENS and visual stimulation in a single solution could enhance the pain-relieving effect of TENS, which has the potential to improve the effectiveness of current pain management treatments.

**Trial Registration:**

Chinese Clinical Trial Registry ChiCTR2500098834; https://www.chictr.org.cn/showprojEN.html?proj=254171

## Introduction

How to improve the efficacy of existing analgesic strategies has always been a clinical concern. The application of transcutaneous electrical nerve stimulation (TENS) has been widely used for pain management, such as postoperative pain, labor pain, and chronic neck pain [1-3]. Low-intensity and high-frequency (50-100 Hz) electrical pulses, a form of TENS, can activate large-diameter A fibers and inhibit the nociceptive volley transmitted via small-diameter A and C fibers that innervate spatially adjacent skin areas [4]. However, the stand-alone applications of TENS may produce limited analgesic effects [5], necessitating the incorporation of additional pain management strategies.

Virtual reality (VR) is a more recent technology that immerses users in a 3D computer simulation environment [6]. Research indicated that VR could reduce pain through various mechanisms, including offering an effective distraction for users to divert their attention away from pain or regulate their emotions [7,8]. One recent study established a connection between spinal cord stimulation and visual feedback in an immersive VR platform, and they found that the combination of stimulation-induced paresthesia and personalized visual bodily feedback resulted in more potent analgesic effects compared to participants who only had VR [9]. This finding suggested that visuo-tactile stimulation in immersive VR might present an opportunity to optimize analgesic efficacy and enhance the analgesic effects of nerve stimulation, such as the TENS. However, the extent to which visuo-tactile stimulation might improve the analgesic effects of TENS and its underlying neurophysiological mechanisms are still poorly explored.

Electroencephalography (EEG) is a reliable bioassay for evaluating how people experience pain and understanding the underlying neurological processes involved in TENS-induced pain relief [10-12]. For example, Peng et al [11] observed that low-frequency TENS had a long-lasting effect on the brain state, as it increased the alpha oscillations in the primary sensorimotor cortex and enhanced the functional connectivity between S1/M1 and the medial prefrontal cortex. Expanding on the research by Peng et al [11], if the incorporation of immersive VR with TENS (TENS-VR) results in enhanced pain relief, it is expected that the reduction of brain activity evoked by painful stimuli will be more pronounced compared to the use of TENS alone.

In this study, we hypothesized that visuo-tactile stimulation that integrated immersive VR with TENS could enhance the analgesic effects of TENS alone in a model of experimentally induced pain, and we also aimed to explore the brain mechanisms underlying the analgesic effect of this integrated intervention. To achieve our aim, we developed a digital therapeutic platform that integrated TENS and immersive VR into a single solution. This immersive VR platform could be able to record and immerse participants in any 360-degree stereoscopic environment and provide additional visual feedback by manipulating digital scenes in a controlled and realistic manner. Three distinct forms of stimulation were applied to produce analgesia, namely TENS alone, congruent TENS-VR (TENS-ConVR), and incongruent TENS-VR (TENS-InVR). The participants in the TENS-ConVR group experienced spatiotemporal congruency between the visual illumination and the tactile sensations elicited by TENS. However, the participants in the TENS-InVR group had spatiotemporal incongruency.

## Methods

### Participants

Healthy participants were recruited from the local community (Multimedia Appendix 1 [11,13-15]). Participants were told that the aim of the study was “to investigate the neurophysiological and perceptual effects of some electrical stimulation delivered to the skin.”

### Ethical Considerations

The experimental procedures were approved by the institutional review board of the Ethics Committee at the First Affiliated Hospital of Xi'an Jiaotong University (XJTU1AF2025 LSYY-384). For privacy and confidentiality, all data were anonymized before analyzing. All participants signed consent forms including a detailed declaration of study-related benefits and risks before participating in the study. All participants received a compensation of CN ¥200 (~US $27.46) upon completion of the study.

### Experimental Design

**Figure 1:**
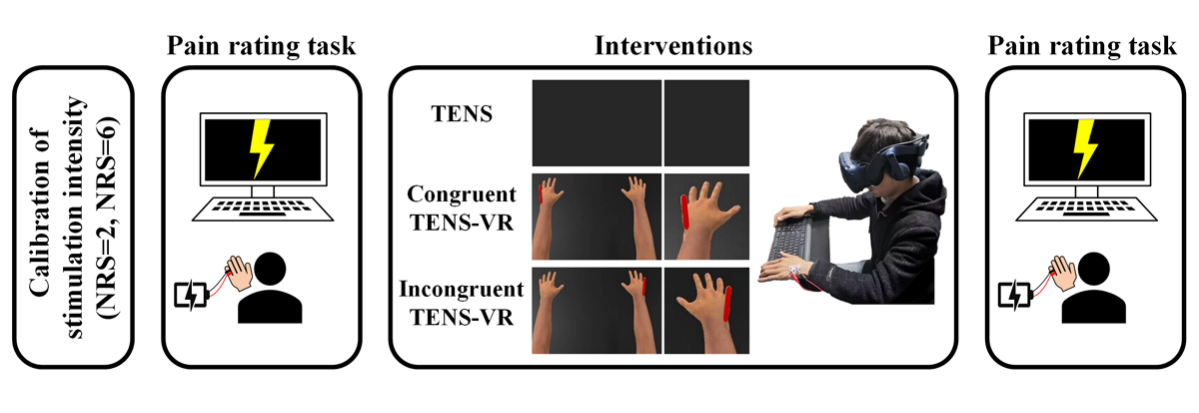
Schematic illustration of the experimental procedure. The experimental procedure included stimulus intensity correction, a pain rating task, an intervention, and a reperformed pain rating task. NRS: numerical rating scale; TENS: transcutaneous electrical nerve stimulation; TENS-ConVR: congruent virtual reality with transcutaneous electrical nerve stimulation; TENS-InVR: incongruent virtual reality with transcutaneous electrical nerve stimulation.

The experiment consisted of 4 stages conducted on a single day: stimulus intensity correction, a pain rating task, an intervention, and a reperformed pain rating task (Figure 1). Two researchers participated in the experiment: one served as the assessor, while the other administered the intervention. To mitigate bias, the outcome assessor was blinded to the intervention conditions.

Using a constant-current electrical stimulator to deliver nociceptive stimuli via electrodes on the participant’s left little finger (Multimedia Appendix 1), the stimulus intensity is calibrated using an ascending method until the participant rates the intensity of pain as 2 and 6 on a numerical rating scale (NRS; from 0=no pain to 10=unbearable pain). Low-intensity (NRS=2) and high-intensity (NRS=6) electrical stimuli were used in the pain rating task. The determination of the thresholds for low-intensity and high-intensity electrical stimuli is grounded in research conducted by Peng et al [11].

The pain rating task was used to assess the subjective perception of pain in response to pain-inducing stimuli. Participants experienced 2 distinct levels of physical stimulation: low-intensity (NRS=2) and high-intensity (NRS=6). The experiment comprised 4 sessions. Each session consisted of 15 trials, with 10 high-intensity trials and 5 low-intensity trials. These trials were presented in a pseudorandomized order. Each trial began with an 800-1200 ms fixation cross, and participants received either a low-intensity or high-intensity stimulus to the left little finger through a pair of ring electrodes (duration=50 ms). Participants then rated the intensity of the perceived pain on the 0-10 NRS for 2000 ms. The intertrial interval was 8000 ms.

All participants were randomly assigned to receive 1 of 3 interventions: TENS alone, TENS-ConVR, or TENS-InVR. After the pain rating task, participants received a 30-minute intervention according to the randomized stimulation protocols. TENS was generated by a constant current electrical stimulator (Sanxia Technique Inc) and delivered through a pair of surface round electrodes (diameter: 16 mm; interelectrode distance: 3 cm) placed over the ulnar nerve on the dorsum of the left hand. The frequency of stimulation was 85 Hz [3], and the pulse width was 200 µs. The duration of the TENS session was 30 minutes and was divided into 5 blocks. Each block was stimulated for 5 minutes and rested for 1 minute. The stimulus intensity was individually adjusted to elicit a strong but nonpainful tingling sensation (Multimedia Appendix 1).

In order to test whether visuo-tactile stimulation from TENS-ConVR could enhance the analgesic effects of TENS alone, we developed an immersive digital environment referred to as the “VR pain relief experiment platform.” A neutral physiotherapy environment was established, comprising a small room with no additional things that could interfere with the experiment. Participants entered the scene using an HTC Vive Pro headset (2880×1600 display resolution; 110 field of view; 90 Hz refreshing rate; and head orientation tracking gravity sensor, gyroscope, magnetometer, and constellation tracking camera), while also being connected to electrical stimulation devices.

The digital arm positions were aligned with respect to the participant’s real physical arms. Initially, participants were instructed to identify the area of the hand where they experienced tactile sensations from the TENS. Then, the defined region was illuminated by adjusting visual parameters until it was closely aligned with the subjective experience reported by the participants. In the TENS group, participants observed a dark screen in the immersive digital environment (Figure 1). In the TENS-ConVR group, the immersive TENS-VR platform illuminated the area according to the sensations the individual experienced as induced by TENS (Figure 1). In the TENS-InVR group, VR illuminated the area of the hand contralaterally to the TENS side in real time (Figure 1). The regions of the digital arm matching the electrical stimulation were highlighted during the 5-minute periods of TENS and not highlighted during the 1-minute breaks, aligning with the changes in the electrical stimulation. Following the completion of the intervention, participants were asked to perform the pain rating task again.

### EEG Data Collection and Processing

During the pain rating task, a 32-channel EEG system was used to record EEG data synchronously (TMSi SAGA; pass band: 1-100 Hz; sampling rate: 1000 Hz). EEG data were preprocessed in the MATLAB environment using EEGLAB, developed by the Swartz Center for Computational Neuroscience at the University of California, San Diego [13]. The details of the data collection and processing can be found in Multimedia Appendix 1.

### Time- and Frequency-Domain Analyses

The event-related potential (ERP) was calculated by averaging the single-trial waveforms in the time domain. The peak latency and amplitude of the N2 and P2 waves were measured from each single-subject average waveform at Cz [11]. Time-frequency distributions of EEG the trials were estimated using a windowed Fourier transform with a fixed 250-ms Hanning window. A frequency-domain analysis was also conducted to evaluate the effect of interventions on spontaneous brain oscillations. Prestimulus EEG signals were extracted from a time window ranging from –1000 ms to 0 ms relative to the stimulus onset (Multimedia Appendix 1).

### Statistical Analysis

Two separate 2-way ANOVAs were conducted to evaluate the effects of different interventions on pain ratings and electrophysiological responses. The first ANOVA compared the TENS-ConVR group with the TENS group, and the second ANOVA compared the TENS-ConVR group with the TENS-InVR group. Each ANOVA involved 2 factors: a between-group factor and a within-participants time factor (before and after intervention). Post hoc comparisons were conducted using 2-tailed paired *t* tests with Bonferroni correction when significant main effects or interactions were observed.

## Results

### Demographics and Questionnaires

**Figure 2:**
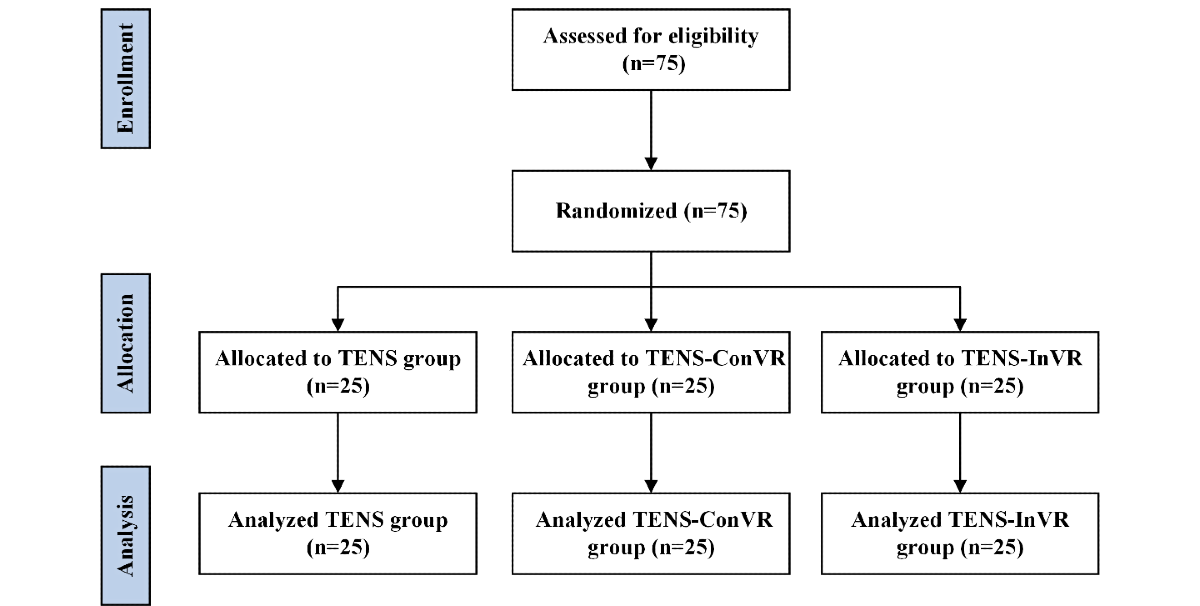
CONSORT (Consolidated Standards of Reporting Trials) flow diagram. TENS: transcutaneous electrical nerve stimulation; TENS-ConVR: congruent virtual reality with transcutaneous electrical nerve stimulation; TENS-InVR: incongruent virtual reality with transcutaneous electrical nerve stimulation.

A priori power analysis using G*Power software (University of Kiel) [16] indicated that 66 participants were required to achieve a statistical power of 0.95 with an α value of .05 for repeated measures ANOVA designs. To account for potential dropouts or errors, a total of 75 healthy, right-handed participants (mean age 21.52, SD 0.26 years) who never had TENS before were recruited (Figure 2). No significant between-group differences (TENS, TENS-ConVR, and TENS-InVR groups) in demographics were found (Table 1; all *P*>.05). No participants reported any adverse effects related to the experiment.

**Table 1:** The comparison of demographic and psychometrical measurements for TENS^a^, TENS-ConVR^b^, and TENS-InVR^c^ groups^d^.

			TENS (n=25)		TENS-ConVR (n=25)		TENS-InVR (n=25)		Statistics		*P* value
**Age (years), mean (SEM)**			21.84 (0.51)		21.48 (0.43)		21.24 (0.42)		0.44 (2, 72)^e^		.64
**Sex, n (%)**									0.33 (2)^f^		.85
	Female	10 (40)		12 (48)		11 (44)					
	Male	15 (60)		13 (52)		14 (56)					
**Weight (kg)** **, mean (SEM)**			63.50 (1.75)		62.38 (1.81)		65.97 (3.48)		0.57 (2, 72)^e^		.57
**Height (cm)** **, mean (SEM)**			172.60 (1.49)		170.40 (1.69)		170.40 (1.53)		0.57 (2, 72)^e^		.57
**Low intensity (µA), mean (SEM)**			1171 (74.23)		1191 (126.40)		1376 (110.20)		0.99 (2, 72)^e^		.38
**High intensity (µA), mean (SEM)**			3074 (267.7)		2915 (275.60)		3444 (253.50)		0.91 (2, 72)^e^		.41

^a^TENS: transcutaneous electrical nerve stimulation.

^b^TENS-ConVR: congruent virtual density with transcutaneous electrical nerve stimulation.

^c^TENS-InVR: incongruent virtual density with transcutaneous electrical nerve stimulation.

^d^Data are expressed using mean (SEM). Statistics were obtained by applying 1-way ANOVA or chi-square test with factors of group (TENS, TENS-ConVR, and TENS-InVR).

^e^*F* test (*df*).

^f^Chi-square (*df*).

### Effect of TENS, TENS-ConVR, and TENS-InVR on Pain Rating

**Figure 3:**
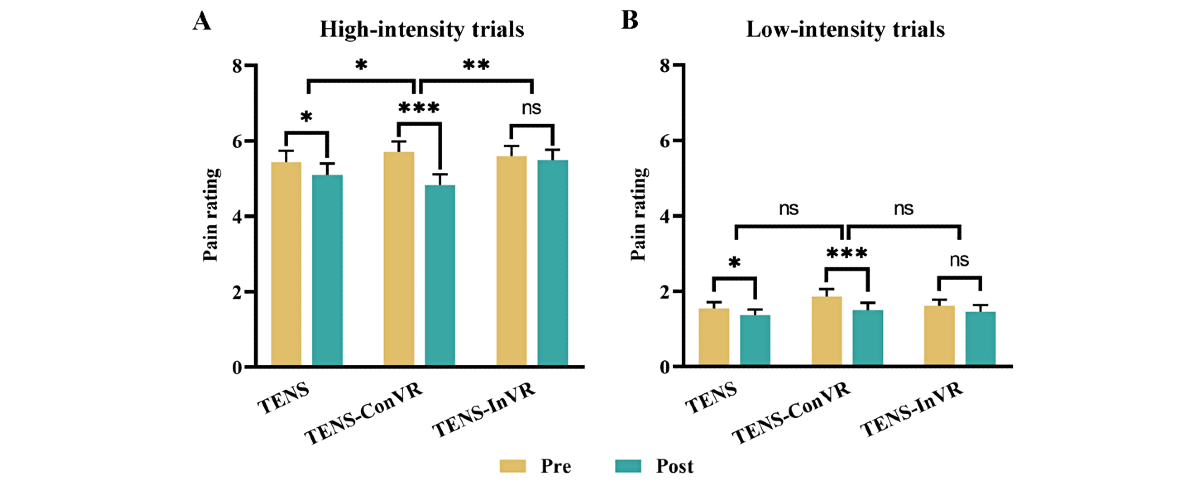
Effect of TENS, TENS-ConVR, and TENS-InVR on subjective pain rating. (A) Subjective pain rating before and after interventions in high-intensity trials. (B) Subjective pain rating before and after interventions in low-intensity trials. The error bar represents SEM. *P<.05; **P<.01, ***P<.001. ns: not significant; TENS: transcutaneous electrical nerve stimulation; TENS-ConVR: congruent virtual reality with transcutaneous electrical nerve stimulation; TENS-InVR: incongruent virtual reality with transcutaneous electrical nerve stimulation.

For high-intensity trials, significant interactions were observed between the “group” and “time” variables in relation to pain rating in ANOVA analysis (TENS-ConVR group vs TENS group and TENS-ConVR group vs TENS-InVR group; Figure 3A). Specifically, participants in the TENS-ConVR groups had a greater pain reduction compared to those in the TENS group (*F*_1,48_=6.84; *P*=.01) and TENS-InVR group (*F*_1,48_=11.54; *P*<.001). After conducting post hoc 2-tailed paired *t* tests on 3 groups, the results showed a significant reduction in pain rating for both the TENS group t_24_=2.32; 95% CI 0.04-0.63; *P*=.03; Cohen *d*=0.46) and the TENS-ConVR group (t_24_=5.84; 95% CI 0.57-1.19; *P*<.001; Cohen *d*=1.17). However, the TENS-InVR group did not exhibit a statistically significant difference in pain intensity before and after intervention (t_24_=0.55; 95% CI –0.27 to 0.46; *P*=.59; Cohen *d*=0.11). For low-intensity trials, no significant interactions were observed between the “group” and “time” when comparing the TENS-ConVR group with the TENS group (*F*_1,48_=1.72; *P*=.20) or the TENS-ConVR group with the TENS-InVR group (*F*_1,48_=2.08; *P*=.16; Figure 3B). In the TENS alone group, no significant difference was observed between high-intensity and low-intensity trials (*F*_1,48_=0.33; *P*=.57; Figure S1A in Multimedia Appendix 2). However, TENS-ConVR– induced pain relieving for high-intensity trials was greater than that for low-intensity trials (*F*_1,48_=8.78; *P*=.005; Figure S1B in Multimedia Appendix 2).

### Effect of Different Interventions on ERP

**Figure 4:**
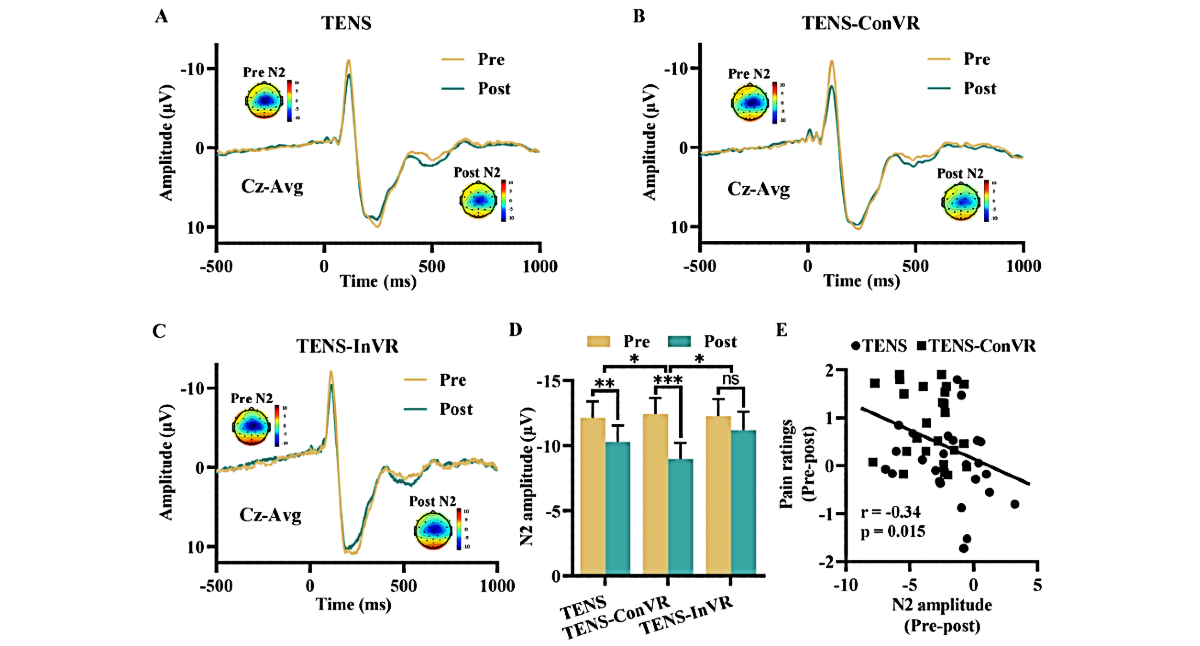
Effect of TENS, TENS-ConVR, and TENS-InVR on event-related potential in high-intensity trials. (A-C) Electrical stimulation-evoked responses in the time domain at the group level. For each experimental group, group-level waveforms and scalp topographies of N2 waves (Cz-Avg) are exhibited. At the peak latency of the N2 waves, scale topographies are displayed. (D) N2 amplitude before and after interventions. (E) Correlations between the changes in N2 amplitude and pain rating across TENS and TENS-ConVR conditions. The error bar represents SEM. *P<.05; **P<.01, ***P<.001. ns: not significant; TENS: transcutaneous electrical nerve stimulation; TENS-ConVR: congruent virtual reality with transcutaneous electrical nerve stimulation; TENS-InVR: incongruent virtual reality with transcutaneous electrical nerve stimulation.

Figure 4 depicts the time-domain scalp morphologies of the ERP waveforms and the N2 pain waves before and after different interventions in high-intensity trials (Figure 4A-C). Significant interactions were observed between the “group” and “time” variables in relation to N2 amplitude in ANOVA analysis (TENS-ConVR group vs TENS group and TENS-ConVR group vs TENS-InVR group; Figure 4D). Specifically, participants in the TENS-ConVR group decreased N2 amplitude more when compared to those in the TENS group (*F*_1,48_=5.69; *P*=.02) and the TENS-InVR group (*F*_1,48_=5.41; *P*=.02). After conducting post hoc 2-tailed paired *t* tests on 3 groups, the results showed a significant reduction in N2 amplitude for both the TENS group (t_24_=3.53; 95% CI –2.93 to –0.77: *P*=.002; Cohen *d*=0.71) and the TENS-ConVR group (t_24_=8.37, 95% CI –4.29 to –2.59; *P*<.001; Cohen *d*=1.67). However, the TENS-InVR group did not exhibit a statistically significant difference in N2 amplitude before and after intervention (t_24_=1.19, 95% CI –3 to 0.8; *P*=.25; Cohen *d*=0.24). For P2 amplitude, no significant interactions were observed between the “group” and “time” when comparing the TENS-ConVR group with the TENS group (*F*_1,48_=0.04; *P*=.85) or the TENS-ConVR group with the TENS-InVR group (*F*_1,48_=0.11; *P*=.74; Figure S2 in Multimedia Appendix 2). A significant negative correlation was found between the changes in N2 amplitude and pain ratings (*r*=–0.34; *P*=.02; Figure 4E). The time-domain scalp morphologies of the ERP waveforms and the N2 pain waves, both before and after different interventions in low-intensity trials, are detailed in Figure S3A-C in Multimedia Appendix 2. Our analysis found no significant interactions between the “group” and “time” variables in N2 amplitude (TENS-ConVR group vs TENS group: *F*_1,48_=0.002; *P*=.97 and TENS-ConVR group vs TENS-InVR group: *F*_1,48_=1.25; *P*=.27; Figure S3D in Multimedia Appendix 2) or P2 amplitude (TENS-ConVR group vs TENS-group: *F*_1,48_=0.24; *P*=.63 and TENS-ConVR group vs TENS-InVR group: *F*_1,48_=0.002; *P*=.96; Figure S3E in Multimedia Appendix 2).

**Figure 5:**
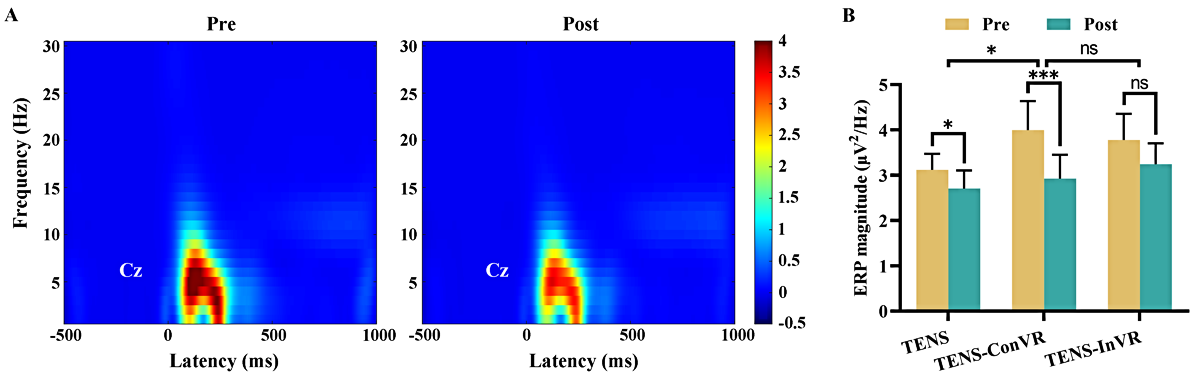
Effect of TENS, TENS-ConVR, and TENS-InVR on time-frequency responses at Cz in high-intensity trials. (A) Group-level time-frequency distributions of ERP responses averaged across intervention groups. (B) ERP magnitude before and after interventions. The error bar represents SEM. *P<.05; **P<.01, ***P<.001. ERP: event-related potential; ns: not significant; TENS: transcutaneous electrical nerve stimulation; TENS-ConVR: congruent virtual reality with transcutaneous electrical nerve stimulation; TENS-InVR: incongruent virtual reality with transcutaneous electrical nerve stimulation.

Figure 5A illustrates group-level, time-frequency distributions in high-intensity trials, with electrical stimuli eliciting a substantial phase-locked response (ERP:100-500 ms, 1-10 Hz). Significant interactions were observed between the “group” (TENS-ConVR and TENS groups) and “time” variables in relation to ERP magnitude in ANOVA analysis (Figure 5B). Specifically, participants in the TENS-ConVR group had a greater reduction in ERP magnitude compared to those in the TENS group (*F*_1,48_=4.85; *P*=.03). Post hoc paired *t* tests showed a significant reduction in ERP magnitude for both the TENS group (t_24_=2.22; 95% CI –0.03 to –0.79; *P*=.04, Cohen *d*=0.44) and the TENS-ConVR group (t_24_=4.54; 95% CI 0.59-1.56; *P*<.001; Cohen *d*=0.91). This result was not observed in the TENS-InVR group (t_24_=1.92; 95% CI –0.04 to 1.11; *P*=.07; Cohen *d*=0.38). The group-level, time-frequency distributions in low-intensity trials have been provided in Figure S4A in Multimedia Appendix 2. Our analysis found no significant interactions between the “group” and “time” variables in relation to ERP magnitude for low-intensity trials (TENS-ConVR group vs TENS group: *F*_1,48_=0.002; *P*=.96 and TENS-ConVR group vs TENS-InVR group: *F*_1,48_=1.25; *P*=.27; Figure S4B in Multimedia Appendix 2).

### Effect of Different Interventions on Spontaneous Oscillations

Figure S5A-C in Multimedia Appendix 2 depicts the EEG spectra of spontaneous oscillations and scalp topographies before and after different interventions. Participants in the TENS-ConVR and TENS-InVR groups showed a statistically significant reduction in gamma amplitude after stimulation (*P*=.02 and *P*=.001, respectively; Figure S5D in Multimedia Appendix 2). No significant pre- and postintervention differences were observed in the remaining frequency bands for all intervention groups (*P*>.05). A significantly negative correlation was observed between the changes in N2 amplitude and gamma amplitude (*r*=–0.33; *P*=.02; Figure S5E in Multimedia Appendix 2).

## Discussion

### Principal Findings

This study presented a digital therapeutic platform that synergistically combines TENS and VR. Our results demonstrated that the combined TENS and VR intervention, referred to as TENS-ConVR, enhanced the pain-relieving effect of TENS. This integrated approach led to a more pronounced decrease in N2 amplitude compared to TENS alone. Interestingly, participants who reported stronger pain relief also showed a greater decrease in brain activity. Additionally, TENS-ConVR was found to reduce gamma amplitude, which was correlated with the decreased N2 amplitude. These findings suggested that the integration of TENS and visual stimulation in a single solution had the potential to improve the effectiveness of current pain management treatments.

Multiple studies have provided evidence that immersive VR could alleviate pain through distraction or mindfulness-based emotional regulation [7,8,17]. This is achieved by immersing individuals in a computer-generated environment with serene landscapes, soothing music, and other elements [8,18]. However, our findings indicated that the enhanced pain-relieving effect of TENS-ConVR cannot be simply attributed to the distracting effects of VR. This is evidenced by the lack of a statistically significant difference in pain intensity reduction in the TENS-InVR condition, where visual stimuli and tactile stimuli were not spatially congruent. Notably, the analgesic effect of TENS-ConVR was found to be more than double the effect observed in the TENS-InVR condition. Therefore, simply exposing participants to VR or incorporating a visual representation of a participant’s body in TENS does not suffice to replicate the pain-relieving effect observed in the TENS-ConVR condition. Notably, we observed an enhanced pain-relieving effect of TENS-ConVR only in high-intensity trials, not in low-intensity trials. Consistent with this, previous studies indicated that VR-based distraction might be more effective at higher levels of pain intensity [19,20]. Therefore, we speculated that the increased pain relief observed with TENS-ConVR may be associated with pain stimuli intensity. Additionally, using a 2-point NRS may lack the sensitivity required to detect minor changes for TENS-ConVR, potentially explaining the absence of an enhanced pain-relieving effect in low-intensity trials.

Studies have demonstrated that pain perception is susceptible to the influence of multisensory inputs and the integration of various body signals [21-23]. This perception is closely tied to any abnormalities in the central representation of painful body parts [24]. The use of congruent multisensory stimulation has the potential to modify this central body representation, thereby providing potential pain relief [9,25]. In a study conducted by Solcà et al [25], participants were shown their digital hands flashing in synchrony or asynchrony with their detected heartbeat. The study found that when the flashing was congruent, it led to reduced pain and increased hand strength in patients with complex regional pain syndrome. This effect was not observed when the flashing was incongruent. Our findings are consistent with the above results. We speculated that the congruent combination of visual and tactile stimulation in the TENS-ConVR condition may foster a sense of ownership over the digital body. This could potentially influence the central representation of the body and consequently enhance the pain-relieving effects of TENS.

Notably, the enhanced pain-relieving effect of TENS-ConVR was further supported through the ERP findings. It showed a significantly reduced N2 amplitude in the TENS-ConVR condition compared to both TENS alone and TENS-InVR conditions. The N2 component, triggered by painful stimuli and indicating neuronal activation in both the operculo-insular cortices and the contralateral primary somatosensory cortex, is believed to be associated with the processing of pain [26]. According to the gate control theory of pain, applying low-intensity, non-noxious TENS could activate Aβ fibers, thereby inhibiting the transmission of nociceptive signals [27]. Recent studies have used nociceptive stimulation to investigate the analgesic effect of TENS. Researchers measured perceptual and cerebral responses and observed a reduction in N2 amplitude in the active TENS group compared to the sham TENS group [11]. We speculated that the reduced N2 amplitude in the TENS-ConVR condition may be attributed to the congruent visuo-tactile stimulation, which could drive a coherent body representation and potentially enhance somatosensory inhibitory interactions. The discovery of a statistically significant positive connection between the reduction in pain ratings and the decrease in N2 amplitude may provide validity to our inference.

Spontaneous EEG gamma oscillations have been extensively investigated in cognitive processes involved in perception and attention [28]. A study by Hauck et al [29] examined the impact of directed attention on pain-related oscillations and synchronization processes. They found a pronounced increase in induced oscillatory activity within the gamma frequency band when participants focused their attention on painful stimuli. In our results, we found that TENS-ConVR reduced spontaneous gamma oscillations. Additionally, a negative correlation between changes in N2 amplitude and gamma amplitude was observed. We speculated that the reduced spontaneous gamma oscillations may be attributable to decreased attention to pain, facilitated by the multisensory information presented in the immersive VR condition.

TENS is a cost-effective, nonpharmacological intervention that has been widely used for pain management [30]. However, repeated application of TENS has been shown to result in analgesic tolerance within several days in both animal and human studies [31,32]. Unlike other additional therapies for TENS [33-35], such as the combining TENS with local heat and cold applications [35], TENS-VR minimizes the risk of allodynia, a condition where movement or even gentle touch can increase or induce pain, reported by many patients with chronic pain [36]. Furthermore, our digital immersive VR platform enables automatized integration with existing pain treatments, minimizing the active involvement of both patient and therapist. It could enable the implementation of prolonged and repeated stimulation in future TENS-VR investigations, potentially improving its effects.

### Limitations

This study has several limitations that should be acknowledged. First, the simplicity of the VR scenario may not adequately induce the desired levels of immersive experience and ownership illusions within the VR environment. Further studies could refine and customize the VR scenario to enhance immersion. Second, the pain relief effect of the TENS-VR was only assessed in healthy participants experiencing experimental pain. Further work is necessary to determine the effectiveness of pain relief in both acute and chronic conditions. Third, there is a weak but statistically significant association between the change in N2 amplitude and the change in NRS score, potentially affected by individual variability and a small sample size. Further studies require validation with a larger sample. Furthermore, the absence of applying a stimulus to the pain threshold and tolerance to assess the effectiveness of TENS, TENS-VR on pain relief represents a limitation in our study. Future studies should include these factors to provide a more comprehensive evaluation of treatment efficacy.

### Conclusions

This study observed that combining TENS and visual stimulation in a single solution could enhance the pain-relieving effect of TENS, which has the potential to improve the effectiveness of current pain management treatments.

## Appendix

Multimedia Appendix 1 Supplementary methods.

Multimedia Appendix 2 Supplementary figures.

Multimedia Appendix 3 CONSORT 2010 checklist.

## Abbreviations

CONSORT: Consolidated Standards of Reporting Trials
EEG: electroencephalography
ERP: event-related potential
NRS: numerical rating scale
TENS: transcutaneous electrical nerve stimulation
TENS-ConVR: congruent virtual reality with transcutaneous electrical nerve stimulation
TENS-InVR: incongruent virtual reality with transcutaneous electrical nerve stimulation
TENS-VR: virtual reality with transcutaneous electrical nerve stimulation
