# Supplementary material for: Neurobiological Mechanisms of Enhanced Pain-Relieving Transcutaneous Electrical Nerve Stimulation via Visuo-Tactile Stimulation in Immersive Virtual Reality: Randomized Controlled Trial
Source: J Med Internet Res. 2025 Mar 19;27:e63137. doi: 10.2196/63137 (PMC11966074; doi:10.2196/63137)
Supplement: Multimedia Appendix 1 [file jmir_v27i1e63137_app1.docx]

**Participants**

Participants were excluded if they had peripheral and central nervous system disease, cardiac pacemaker, chronic pain, or if they were under any type of pain medication, medical condition predisposing to nausea or dizziness.

**Stimulus intensity correction**

A constant-current electrical stimulator (Sanxia Technique Inc., China) produced and delivered nociceptive stimuli through a pair of surface electrodes (diameter: 16 mm; inter-electrode distance: 3 cm) placed over the little finger of the left hand. Each stimulus consisted of a 50-ms constant-current square-wave pulse. For each participant, the ascending method of limits (pulses with an ascending current starting at 0.2 mA and increasing in steps of 0.3 mA) was applied in the calibration phase to determine the stimulus intensity, until two ratings of 2 and 6 out of 10 were obtained on a numerical rating scale (NRS), in which 0 indicated no sensation and 10 indicated unbearable pain. Painful (NRS = 6) and nonpainful (NRS = 2) electrical stimuli were used in the pain rating task. Each participant was instructed to verbally report pain scores to assess the intensity of pain perception. This procedure was repeated three times, and the averaged stimulus intensity was calculated for the following experiment. In addition, the averaged stimulus intensity was presented several times in random orders to ensure rating consistency.

**Intervention**

Given that the TENS sensation habituates after the first occurrences of the stimulus, the desired perceptual outcome of each condition was obtained in three consecutive attempts. The stimulus intensity used in the actual experiment was the highest of the three attempts. Before the TENS, all participants were given the following instruction: “You will now receive 30 minutes of TENS, with a one-minute rest for every five minutes of stimulation”.

**EEG data collection and processing**

All participants sat in a comfortable chair in a quiet room with a temperature ranging from 24 to 28 °C. They were instructed to concentrate on the stimuli while keeping their eyes open and looking at a fixation point on the screen. A curtain was used to conceal the subjects’ forearms. The nose was used as the online reference, and the electrode impedance was kept below 10 kΩ.

Continuous EEG data were bandpass filtered between 1 and 30 Hz and re-referenced to the average reference. The EEG epochs were extracted using a window analysis time 1500 ms (500 ms pre-stimulus and 1000 ms post-stimulus), and the baseline was corrected using the pre-stimulus interval. Trials contaminated by eye blinks and movements were corrected using an Independent Component Analysis algorithm [1].

**Time- and frequency-domain analyses**

*Time-domain analysis.* In order to examine the effects of electrical stimulation on EEG signals, the event-related potential (ERP) was calculated by averaging the single-trial waveforms in the time domain. The peak latency and amplitude of the N2 and P2 waves, defined as the most negative and positive deflections between 150 and 500 ms after stimulus onset respectively [2], were measured from each single-subject average waveform at Cz. Single-subject average ERP waveforms were subsequently averaged across subjects to obtain group-level ERP waveforms. The group-level scalp topographies at the peak latency of N2 and P2 waves were generated using spline interpolation.

*Time-frequency analysis.* Time-frequency distributions (TFDs) of EEG the trials were estimated using a windowed Fourier transform (WFT) with a fixed 250-ms Hanning window. WFT yielded, for each trial, a complex time-frequency estimate F(t, f) at each time-frequency point (t, f), extending from -500 ms to 1000 ms (in steps of 1 ms) in the time domain, and from 1 to 30 Hz (in steps of 1 Hz) in the frequency domain. The resulting spectrogram, P(t, f) = |F(t, f)|^2^, represents the signal magnitude as a joint function of time and frequency at each time-frequency point. The spectrogram was baseline-corrected (reference interval: from -375 to -125 ms) at each frequency using the subtraction approach [3]. Magnitudes of ERP at Cz (100-400 ms,1-10 Hz) were calculated by computing the mean of the top 20% time-frequency points for each subject in each intervention group [4].

*Frequency-domain analysis.* For each subject and session, pre-stimulus EEG signals were transformed to the frequency domain using a discrete Fourier transform, yielding an EEG spectrum ranging from 1 to 97 Hz. Single-subject EEG spectra were averaged across subjects to obtain group-level spectra of spontaneous EEG oscillations for each intervention group at Cz.

**References**

1. Delorme A, Makeig S. EEGLAB: an open source toolbox for analysis of single-trial EEG dynamics including independent component analysis. J Neurosci Methods. 2004 Mar 15;134(1):9-21. PMID: 15102499. doi: 10.1016/j.jneumeth.2003.10.009.

2. Peng WW, Tang ZY, Zhang FR, Li H, Kong YZ, Iannetti GD, et al. Neurobiological mechanisms of TENS-induced analgesia. Neuroimage. 2019 Jul 15;195:396-408. PMID: 30946953. doi: 10.1016/j.neuroimage.2019.03.077.

3. Hu L, Xiao P, Zhang ZG, Mouraux A, Iannetti GD. Single-trial time-frequency analysis of electrocortical signals: baseline correction and beyond. Neuroimage. 2014 Jan 1;84:876-87. PMID: 24084069. doi: 10.1016/j.neuroimage.2013.09.055.

4. Mouraux A, Iannetti GD. Across-trial averaging of event-related EEG responses and beyond. Magn Reson Imaging. 2008 Sep;26(7):1041-54. PMID: 18479877. doi: 10.1016/j.mri.2008.01.011.
