# Supplementary material for: Neurobiological Mechanisms of Enhanced Pain-Relieving Transcutaneous Electrical Nerve Stimulation via Visuo-Tactile Stimulation in Immersive Virtual Reality: Randomized Controlled Trial
Source: J Med Internet Res. 2025 Mar 19;27:e63137. doi: 10.2196/63137 (PMC11966074; doi:10.2196/63137)
Supplement: Multimedia Appendix 2 [file jmir_v27i1e63137_app2.docx]

**Supplementary figures**


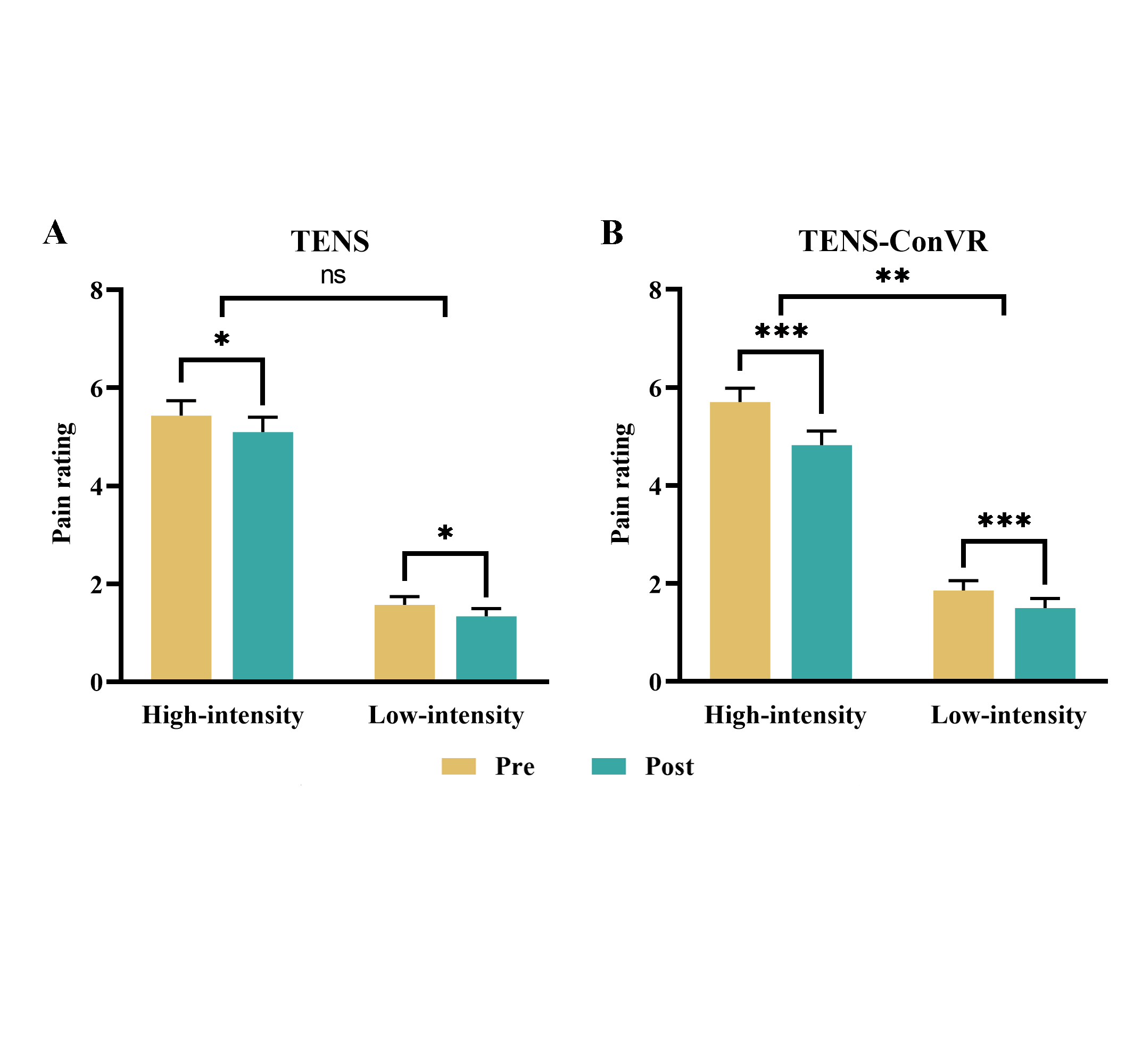


**Figure S1.** Effect of TENS and TENS-ConVR on subjective pain rating in high-intensity trials and low-intensity trials. (A) Subjective pain rating before and after TENS in high-intensity trials and low-intensity trials. (B) Subjective pain rating before and after TENS-ConVR in low-intensity trials. The error bar represents SEM. ns: not significant; TENS: transcutaneous electrical nerve stimulation; TENS-ConVR: congruent virtual reality with TENS. **P*<.05; ***P*<.01, ****P*<.001.


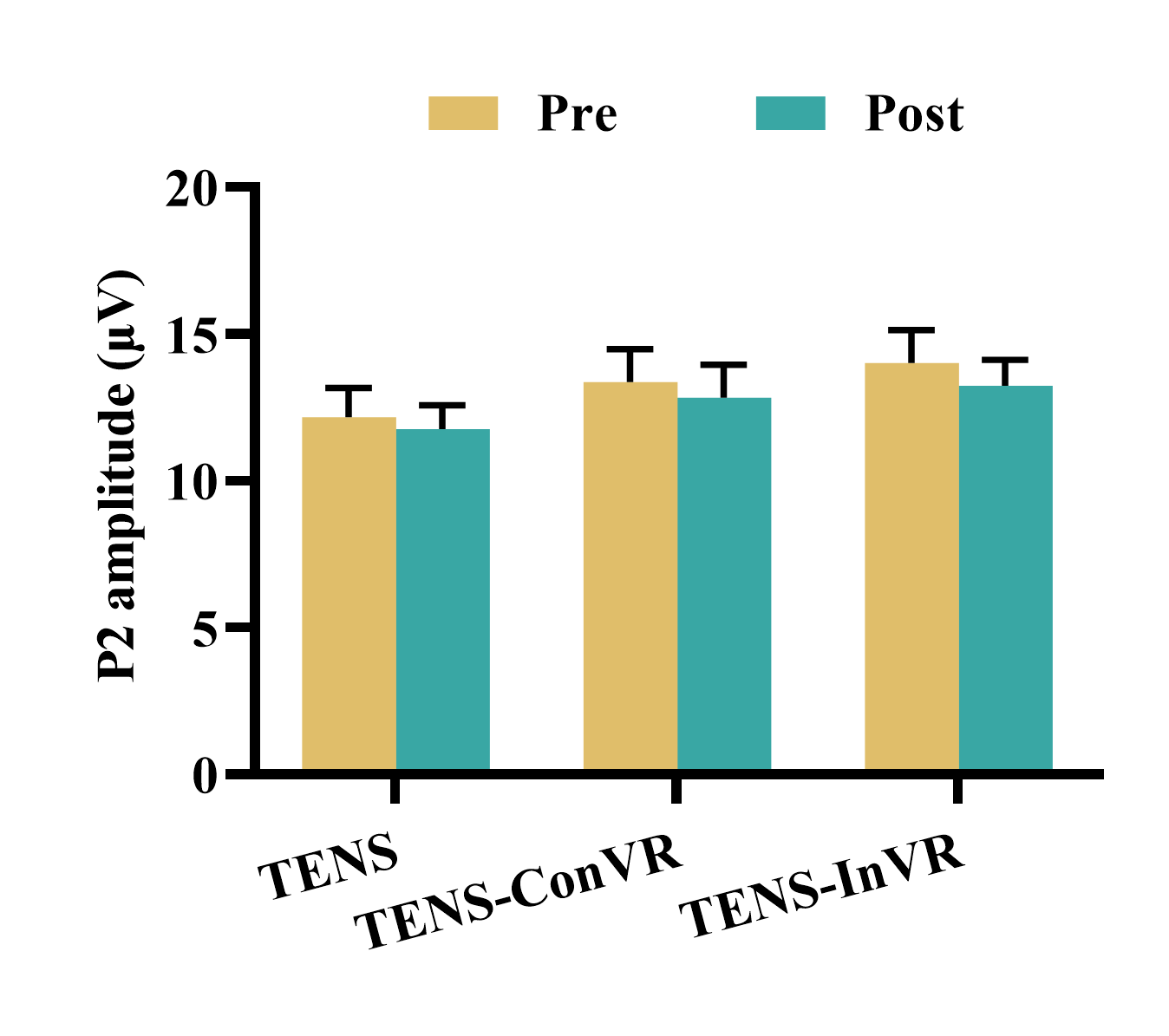


**Figure S2.** P2 amplitude before and after interventions in high-intensity trials. The error bar represents SEM. TENS: transcutaneous electrical nerve stimulation; TENS-ConVR: congruent virtual reality with TENS; TENS-InVR: incongruent virtual reality with TENS.


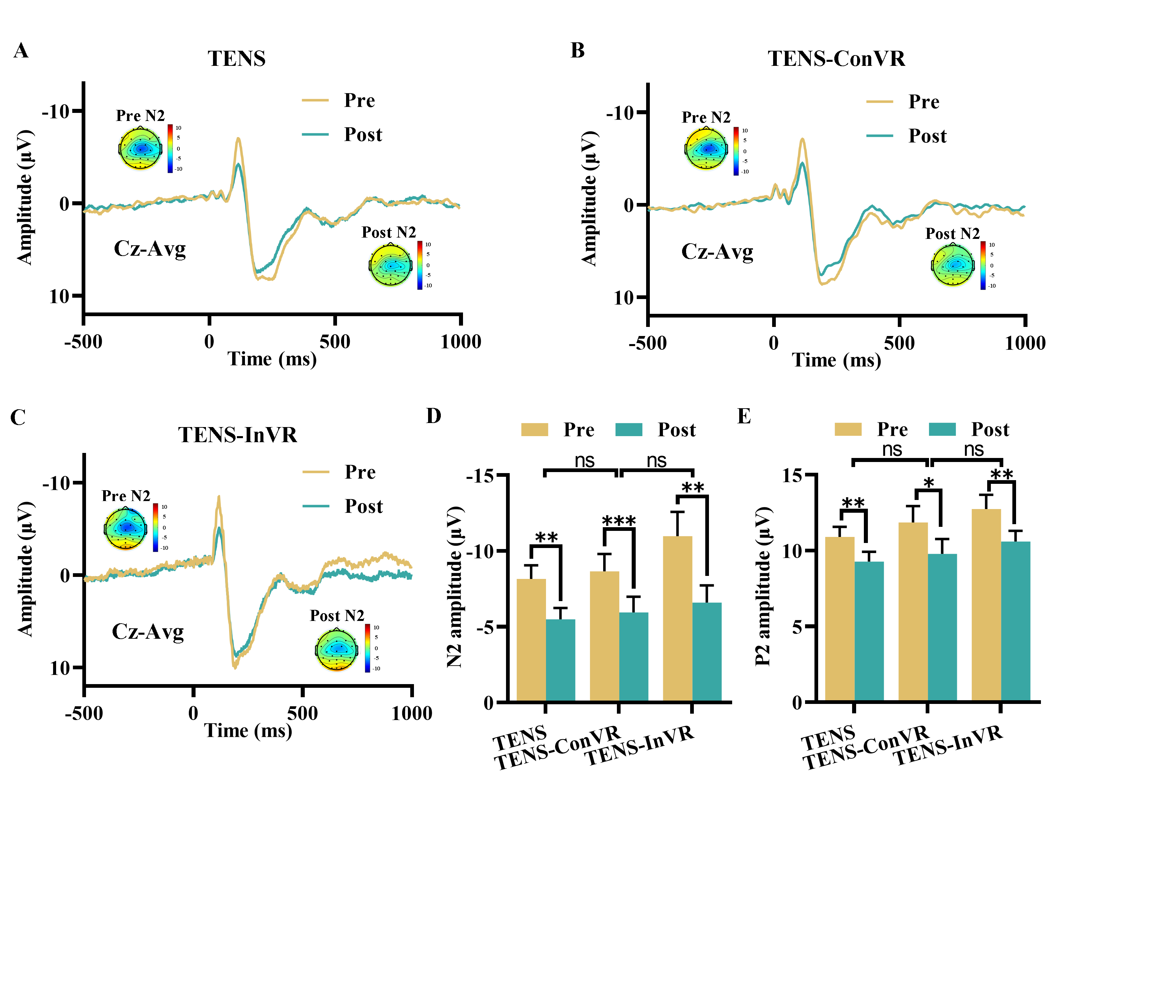


**Figure S3.** Effect of TENS, TENS-ConVR, and TENS-InVR on event-related potential in low-intensity trials. (A-C) Electrical stimulation-evoked responses in the time domain at the group level. For each experimental group, group-level waveforms and scalp topographies of N2 waves (Cz-Avg) are exhibited. At the peak latency of the N2 waves, scale topographies are displayed. (D) N2 amplitude before and after interventions. (E) P2 amplitude before and after interventions. The error bar represents SEM. ns: not significant; TENS: transcutaneous electrical nerve stimulation; TENS-ConVR: congruent virtual reality with TENS; TENS-InVR: incongruent virtual reality with TENS. **P*<.05; ***P*<.01, ****P*<.001.


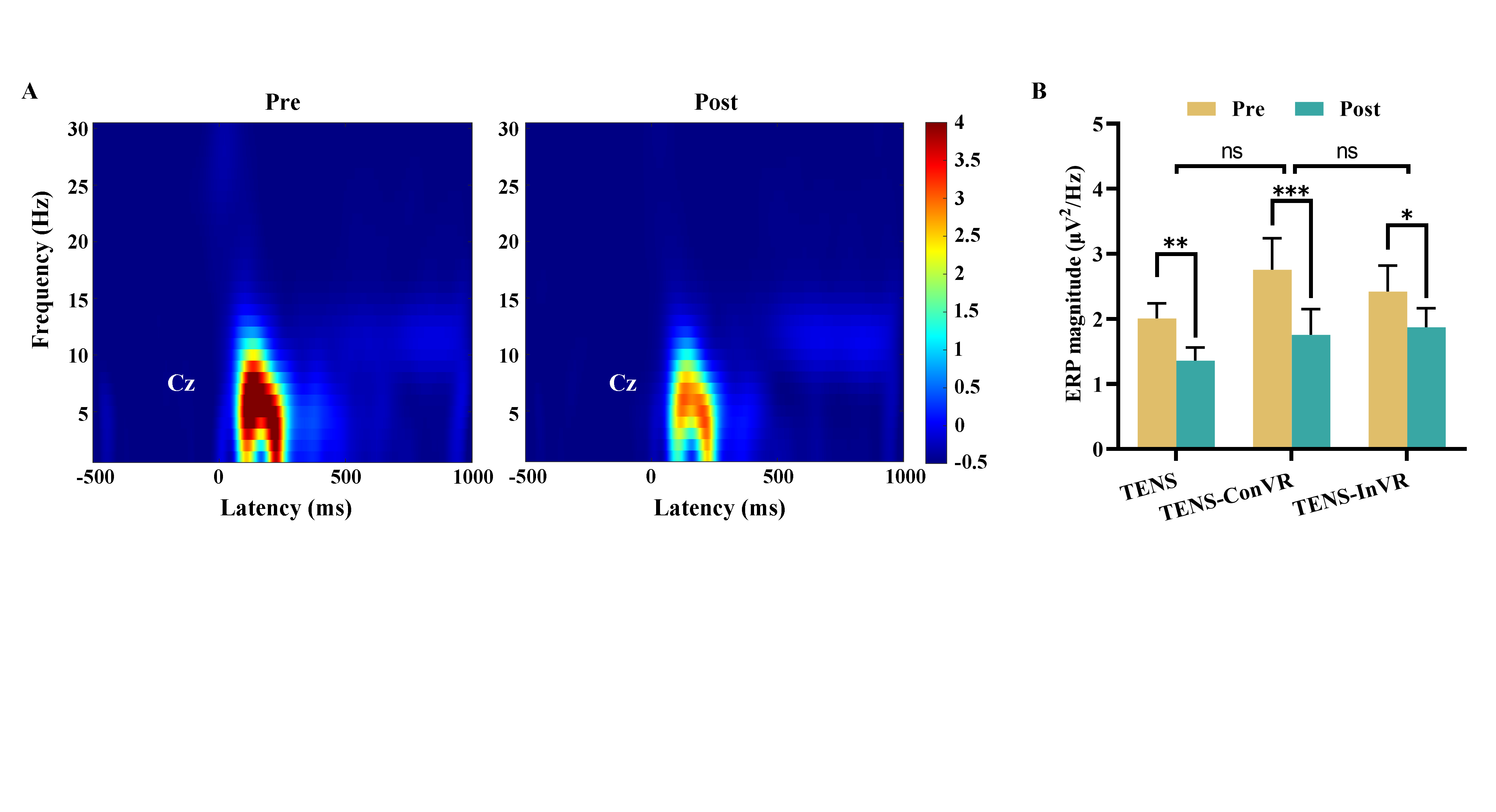


**Figure S4.** Effect of TENS, TENS-ConVR, and TENS-InVR on time-frequency responses at Cz in low-intensity trials. (A) Group-level time-frequency distributions of ERP responses, averaged across intervention groups. (B) ERP magnitude before and after interventions. The error bar represents SEM. ns: not significant; TENS: transcutaneous electrical nerve stimulation; TENS-ConVR: congruent virtual reality with TENS; TENS-InVR: incongruent virtual reality with TENS. **P*<.05; ***P*<.01, ****P*<.001.


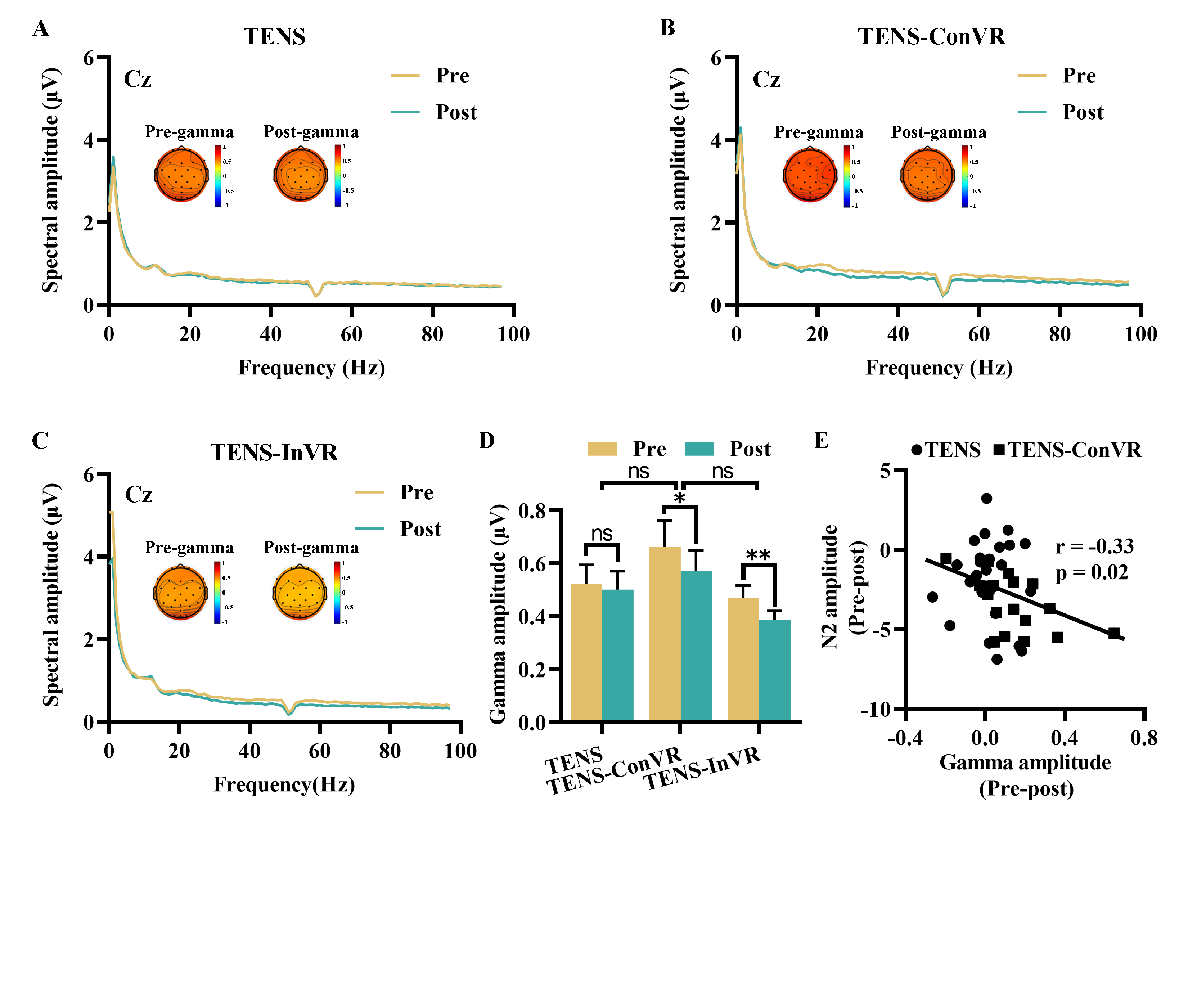


**Figure S5.** Effect of TENS, TENS-ConVR, and TENS-InVR on spontaneous oscillations in high-intensity trials. (A-C) For each experimental group, the amplitude of spontaneous EEG oscillations and the scalp topographies of gamma oscillations (Cz) at the group level are exhibited. (D) Gamma amplitude before and after interventions. (E) Correlations between the changes in N2 amplitude and gamma amplitude across TENS and TENS-ConVR conditions. The error bar represents SEM. ns: not significant; TENS: transcutaneous electrical nerve stimulation; TENS-ConVR: congruent virtual reality with TENS; TENS-InVR: incongruent virtual reality with TENS. **P*<.05; ***P*<.01, ****P*<.001.
